# Drug suicide: a sex-equal cause of death in 16 European countries

**DOI:** 10.1186/1471-2458-11-61

**Published:** 2011-01-29

**Authors:** Airi Värnik, Merike Sisask, Peeter Värnik, Jing Wu, Kairi Kõlves, Ella Arensman, Margareth Maxwell, Thomas Reisch, Ricardo Gusmão, Chantal van Audenhove, Gert Scheerder, Christina M van der Feltz-Cornelis, Claire Coffey, Maria Kopp, Andras Szekely, Saska Roskar, Ulrich Hegerl

**Affiliations:** 1 Estonian-Swedish Mental Health and Suicidology Institute; Estonian Centre of Behavioural and Health Sciences, Õie 39, Tallinn, 11615, Estonia; 2 Institute of Social Work, Tallinn University, Narva mnt 25, Tallinn, 10120, Estonia; 3 The Estonian Institute for Population Studies, Tallinn University, Narva mnt 25, Tallinn, 10120, Estonia; 4 National Suicide Research Foundation, 1 Perrott Avenue, College Road, Cork, Ireland; 5 Department of Nursing and Midwifery, University of Stirling, FK9 4LA, Stirling, Great Britain; 6 Department of Psychiatry, University Hospital of Psychiatry, Bolligenstr.111, 3000 Bern 60, Switzerland; 7 De partamento de Saúde Mental, Faculdade de Ciências Médicas, FCM, Universidade Nova de Lisboa, Campo dos Mártires da Pátria 130, 1169-056 Lisboa, Portugal; 8 Katholieke Universiteit Leuven, LUCAS, Kapucijnenvoer 39 - bus 5310, 3000 Leuven, Belgium; 9 Trimbos-instituut/Netherlands Institute of Mental Health and Addiction, Da Costakade 45, 3521 VS Utrecht, The Netherlands; 10 Institute of Behavioural Sciences, Semmelweis University, Nagyvárad tér 4, 1089 Budapest, Hungary; 11 Inštitut za varovanje zdravja RS Insititute of Public Health of the Republic of Slovenia, Trubarjeva, 1000 Ljubljana, Slovenia; 12 Department of Psychiatry, University of Leipzig, Semmelweisstr.10, 04103 Leipzig, Germany

## Abstract

**Background:**

There is a lack of international research on suicide by drug overdose as a preventable suicide method. Sex- and age-specific rates of suicide by drug self-poisoning (ICD-10, X60-64) and the distribution of drug types used in 16 European countries were studied, and compared with other self-poisoning methods (X65-69) and intentional self-injury (X70-84).

**Methods:**

Data for 2000-04/05 were collected from national statistical offices. Age-adjusted suicide rates, and age and sex distributions, were calculated.

**Results:**

No pronounced sex differences in drug self-poisoning rates were found, either in the aggregate data (males 1.6 and females 1.5 per 100,000) or within individual countries. Among the 16 countries, the range (from some 0.3 in Portugal to 5.0 in Finland) was wide. 'Other and unspecified drugs' (X64) were recorded most frequently, with a range of 0.2-1.9, and accounted for more than 70% of deaths by drug overdose in France, Luxembourg, Portugal and Spain. Psychotropic drugs (X61) ranked second. The X63 category ('other drugs acting on the autonomic nervous system') was least frequently used. Finland showed low X64 and high X61 figures, Scotland had high levels of X62 ('narcotics and hallucinogens, not elsewhere classified') for both sexes, while England exceeded other countries in category X60. Risk was highest among the middle-aged everywhere except in Switzerland, where the elderly were most at risk.

**Conclusions:**

Suicide by drug overdose is preventable. Intentional self-poisoning with drugs kills as many males as females. The considerable differences in patterns of self-poisoning found in the various European countries are relevant to national efforts to improve diagnostics of suicide and appropriate specific prevention. The fact that vast majority of drug-overdose suicides came under the category X64 refers to the need of more detailed ICD coding system for overdose suicides is needed to permit better design of suicide-prevention strategies at national level.

## Background

Choice of suicide method is influenced by such factors as availability of means, cultural acceptance, suicidal intent and individual preference [1-3]. The methods chosen have a major bearing on differential outcomes of suicidal acts. Lethal methods (firearms, drowning and hanging) predominantly characterise suicide while less lethal ones (cutting and poisoning) are often used for suicide attempts [1,4-6]. Females' preferred method is drug overdose, which is not usually lethal, whereas males tend to prefer more lethal methods [2,5]. Methods thus partially explain the sex paradox: although females are more commonly diagnosed as depressed and their suicide attempts are registered more frequently [6,7], males' suicide rates are considerably higher in most countries [8].

Research into suicide methods has mostly explored drug self-poisoning as one of several method categories [1,9,10]. Use of specific drugs has prompted proposals to restrict their availability [11-18]. Some studies have observed an association between rates of suicide and/or attempted suicide and sale of specific medicines or classes of drugs [14-16,19-21]. Studies exploring other forms of self-poisoning, such as charcoal-burning [22] and pesticide ingestion [23,24] focusing mainly on Asian countries, have helped somewhat in formulation of medical management guidelines. Comparative European research on suicidal self-poisoning acts using drugs is lacking.

Previous studies based on data from member countries in the 'European Alliance Against Depression' (EAAD) [25,26] have shown that self-poisoning is the second or third most frequent suicide method for females and males alike. It accounts for a quarter of all female suicides overall and almost half of all female suicides in some countries studied, including Finland, Iceland, England and Scotland [10].

Our study examined rates of sex- and age-specific self-poisoning suicide (ICD-10, X60-64) and determined the category composition of drugs used in 16 European countries. Comparisons were made with other means of self-poisoning (X65-69) and intentional self-harm (X70-84).

## Methods

### Data collection

Data for 2000-04/2005 were collected from 16 member countries in the EAAD project funded by the European Commission. Since data on method-specific suicides are not available from the WHO databank, male and female suicide numbers in 10-year age groups and the respective population data were compiled from the participants' national statistical offices. For the UK, English and Scottish data were collected separately. Belgium is represented by Flanders. A detailed description of the data sources is given elsewhere [9,10].

Data based on the *International Statistical Classification of Diseases and Related Health Problems, Tenth Revision *(ICD-10, WHO 1992) were available from Belgium, Estonia, Finland, France, Germany, Hungary, Iceland, Luxembourg, the Netherlands, Portugal, Scotland, Slovenia, Spain and Switzerland. Self-poisoning with drugs and other substances (codes X60-64 and X65-69) and self-harm (X70-84) were analysed separately. Fatal self-poisoning was identified using ICD-9 (WHO 1978), codes E950-E959, for England in 2000, Portugal in 2000-01 and Ireland for the entire study period. Table 1 lists the five ICD-10 categories of intentional self-poisoning with drugs. No category breakdown was possible for Ireland because the relevant ICD-9 and ICD-10 classifications were not comparable.

**Table 1:** Male and female age adjusted suicide rates per 100 000 and rate ratios, distributed by poisonings and injuries in 16 European countries, means of the years 2000-2004/5

	POISONINGS						INJURIES			TOTAL		
	**Poisoning by drugs X60-X64**				**Other poisonings X65-X69**						**X60-X84**	

	**rate**		**ratio M/F**	**rate**		**ratio M/F**	**rate**		**ratio M/F**	**rate**		**ratio M/F**

	**Male**	**Female**		**Male**	**Female**		**Male**	**Female**		**Male**	**Female**	

Belgium	1.8	1.6	1.1	1.0	0.3	3.3	22.6	7.2	3.2	25.3	9.1	2.8
England	1.3	1.0	1.3	1.1	0.1	8.6	6.8	1.4	4.7	9.2	2.6	3.6
Estonia	0.7	0.9	0.8	1.0	0.2	5.5	41.6	7.1	5.9	43.4	8.2	5.3
Finland	5.3	4.7	1.1	1.9	0.1	15.2	23.7	4.6	5.1	30.9	9.5	3.3
France	2.3	2.4	1.0	0.6	0.3	2.2	22.8	5.9	3.9	25.7	8.5	3.0
Germany	1.5	1.3	1.1	0.7	0.2	4.0	15.4	4.0	3.8	17.6	5.5	3.2
Hungary	3.0	2.9	1.1	2.3	0.7	3.2	39.0	7.2	5.4	44.2	10.7	4.1
Iceland	4.1	2.9	1.4	1.6	0.8	1.9	12.5	2.4	5.1	18.2	6.1	3.0
Ireland	1.6	1.3	1.2	0.6	0.1	9.6	18.0	3.4	5.2	20.2	4.8	4.2
Luxembourg	1.8	1.9	0.9	1.7	0.6	3.1	18.2	4.2	4.4	21.8	6.7	3.3
Netherlands	1.3	1.3	1.0	0.4	0.1	3.3	10.5	4.1	2.6	12.2	5.6	2.2
Portugal	0.3	0.3	0.9	1.8	0.8	2.3	11.3	2.4	4.8	13.3	3.4	3.9
Scotland	3.1	2.6	1.2	1.2	0.1	19.8	13.5	3.0	4.5	17.8	5.7	3.1
Slovenia	0.9	0.9	0.9	2.9	0.6	4.9	37.2	9.4	4.0	41.0	11.0	3.7
Spain	0.4	0.3	1.3	0.5	0.2	2.4	10.0	2.7	3.7	10.8	3.2	3.4
Switzerland	3.0	3.3	0.9	0.8	0.2	3.4	19.9	6.0	3.3	23.7	9.6	2.5
Total	1.6	1.5	1.1	0.8	0.2	3.6	15.6	4.0	3.9	18.0	5.7	3.2

X60 - nonopioid analgesics, antipyretics and antirheumatics

X61 - antiepileptic, sedative-hypnotic, antiparkinsonism and psychotropic drugs, not elsewhere classified

X62 - narcotics and psychodysleptics (hallucinogens), not elsewhere classified

X63 - other drugs acting on the autonomic nervous system

X64 - other and unspecified drugs, medicaments and biological substances

### Data analysis

Annual age- and sex-specific suicide rates were calculated for the 16 countries individually and as a combined EAAD dataset, with age-adjusted rates per 100,000 based on a European standard population [27]. To assess linear correlation (+1 to -1) between national male and female rates of fatal, intentional drug overdose, Pearson correlation coefficients were calculated.

## Results

Overall, annual self-poisoning suicides averaged 2,511 for males and 2,580 for females in 2000-04/2005. As Table 1 shows, the 16 countries' data indicate that male suicides are much more likely to be due to self-harm (including hanging, drowning, shooting and jumping). Male rates of self-poisoning not involving drugs also exceeded female rates in all countries studied. Intentional drug overdoses were almost equally frequent among males and females: 1.6 and 1.5 respectively per 100,000 in the 16 countries combined. Finland had the highest rates for both sexes.

The male-to-female ratio of suicide by drug self-poisoning in the aggregate data was 1.1, ranging from 0.8 (Estonia) to 1.4 (Iceland) in individual countries. For other self-poisoning the aggregate male-to-female ratio was 3.7, and for self-harm 3.9. Males' and females' drug-overdose rates in the countries studied were strikingly similar (r = 0.97). Rates of male and female self-injury (r = 0.88) and self-poisoning not involving drugs (r = 0.67) also showed high positive correlation.

As Table 2 shows, for both sexes in the combined data set, the vast majority of drug-overdose suicides were registered as X64 ('Intentional self-poisoning by and exposure to other and unspecified drugs, medicaments and biological substances'), followed by X61 ('antiepileptic, sedative-hypnotic, antiparkinsonism and psychotropic drugs, not elsewhere classified'). The X63 category ('other drugs acting on the autonomic nervous system') was least frequently used. Category proportions of drugs used for overdose varied widely, but were similar for both sexes in each country. In France, Luxembourg, Portugal and Spain, X64 drugs accounted for more than 70% of overdoses. Finland showed low X64 and high X61 figures. Scotland had high levels of X62 ('narcotics and psychodysleptics [hallucinogens], not elsewhere classified') classification for both sexes, while England exceeded other countries in category X60 ('nonopioid analgesics, antipyretics and antirheumatics').

**Table 2:** Percent distribution of drug poisoning suicides (X60-X64 by ICD-10) in 16 European countries by gender, means of the years 2000-2004/5

	X60		X61		X62		X63		X64		X60-64	
	**Male**	**Female**	**Male**	**Female**	**Male**	**Female**	**Male**	**Female**	**Male**	**Female**	**Male**	**Female**

Belgium	0.0	0.0	30.9	35.8	9.1	3.8	0.0	0.0	60.0	60.4	100.0	100.0
England*	29.8	30.0	21.4	23.2	12.4	8.5	1.2	0.7	35.3	37.5	100.0	100.0
Estonia	0.0	0.0	50.0	42.9	0.0	0.0	0.0	14.3	50.0	42.9	100.0	100.0
Finland	4.2	4.5	72.7	67.9	10.5	10.4	4.9	6.7	7.7	10.4	100.0	100.0
France	0.6	0.4	17.4	17.0	1.5	1.1	1.0	1.1	79.5	80.5	100.0	100.0
Germany	1.1	1.3	24.3	26.8	9.7	3.6	1.4	1.5	63.5	66.8	100.0	100.0
Hungary	1.7	1.4	48.4	55.7	2.5	2.3	2.6	2.0	44.9	38.7	100.0	100.0
Iceland	3.4	5.3	48.3	73.7	0.0	0.0	0.0	5.3	48.3	15.8	100.0	100.0
Luxembourg	0.0	0.0	25.0	40.0	0.0	0.0	0.0	0.0	75.0	60.0	100.0	100.0
Netherlands	2.7	4.2	23.4	28.3	18.0	9.2	0.9	0.8	55.0	57.5	100.0	100.0
Portugal**	0.0	0.0	13.3	21.1	6.7	0.0	0.0	0.0	80.0	78.9	100.0	100.0
Scotland	12.8	14.3	33.3	35.7	43.6	38.6	1.3	2.9	9.0	8.6	100.0	100.0
Slovenia	0.0	0.0	33.3	54.5	33.3	18.2	0.0	0.0	33.3	27.3	100.0	100.0
Spain	0.0	1.5	17.4	22.4	7.0	3.0	1.2	0.0	74.4	73.1	100.0	100.0
Switzerland	0.8	0.6	70.2	77.1	4.1	3.8	5.8	0.0	19.0	18.5	100.0	100.0
Total	5.5	4.9	28.7	31.4	8.5	4.9	1.7	1.4	55.6	57.3	100.0	100.0

X60 - nonopioid analgesics, antipyretics and antirheumatics

X61 - antiepileptic, sedative-hypnotic, antiparkinsonism and psychotropic drugs, not elsewhere classified

X62 - narcotics and psychodysleptics (hallucinogens), not elsewhere classified

X63 - other drugs acting on the autonomic nervous system

X64 - other and unspecified drugs, medicaments and biological substances

The highest rates of drug self-poisoning suicide in the combined dataset were found in males aged 35-54 and females aged 45-54 (Table 3). The 15-24 age group had the lowest rates for both sexes. For males, rates fell off in the 55-64 age group but were slightly elevated among those aged 65+. For females, rates in both the 55-64 and the older (65+) age group were consistently lower.

**Table 3:** Male and female suicide rates per 100 000, poisoning by drugs (X60-X64 by ICD 10) by age groups in 16 European countries, means of 2000-2004/5

			Males							Females			
	**15-24**	**25-34**	**35-44**	**45-54**	**55-64**	**65+**		**15-24**	**25-34**	**35-44**	**45-54**	**55-64**	**65+**

Belgium	1.2	2.5	3.1	3.3	1.9	1.3	Belgium	0.7	1.2	3.2	3.6	2.4	1.3
England	0.5	1.9	2.3	2.3	1.4	1.9	England	0.5	1.1	1.3	1.6	1.6	2.0
Estonia	1.0	0.5	0.9	0.8	1.5	0.2	Estonia	1.2	2.0	0.7	1.0	1.4	0.5
Finland	2.1	7.1	10.8	10.6	5.5	3.8	Finland	2.3	4.3	7.9	9.8	7.9	4.0
France	0.7	3.0	5.0	4.5	2.4	1.7	France	0.7	1.9	4.2	5.1	3.8	2.4
Germany	0.6	1.8	2.4	2.4	2.1	2.1	Germany	0.6	1.0	1.7	2.2	2.3	2.7
Hungary	0.9	2.4	5.3	5.6	3.7	5.9	Hungary	0.6	1.3	3.7	5.5	4.1	7.5
Iceland	1.7	7.3	6.7	8.4	3.1	3.6	Iceland	0.0	2.7	2.9	7.8	6.7	2.4
Ireland	1.1	2.9	2.8	2.4	2.1	1.1	Ireland	1.6	1.0	2.4	2.1	1.7	1.3
Luxembourg	0.6	2.0	3.0	3.7	0.7	3.9	Luxembourg	0.0	0.5	1.8	5.5	4.4	3.1
Netherlands	0.4	1.5	2.1	2.3	1.9	2.0	Netherlands	0.2	0.9	2.0	2.9	2.3	2.1
Portugal	0.2	0.3	0.5	0.5	0.5	0.4	Portugal	0.0	0.3	0.6	0.5	0.6	0.5
Scotland	2.6	5.2	6.0	4.3	2.8	2.1	Scotland	2.7	3.2	4.3	4.7	2.7	1.8
Slovenia	0.8	2.1	1.1	1.2	0.4	0.7	Slovenia	1.0	0.4	1.8	1.2	1.5	1.4
Spain	0.1	0.6	0.8	0.5	0.4	0.3	Spain	0.1	0.3	0.5	0.6	0.4	0.2
Switzerland	0.7	1.7	2.7	3.3	4.3	12.3	Switzerland	1.0	1.6	2.5	3.7	6.4	12.3
Total	0.6	1.9	2.8	2.8	1.9	2.0	Total	0.6	1.2	2.2	2.9	2.4	2.4

Scotland had the highest drug-overdose suicide rates in the 15-24 age group. Among people aged 25-34 Finland, Iceland and Scotland had the highest rates. For the 65+ age group, remarkably high rates of suicide by drug self-poisoning were found in Switzerland (females 12.31, males 12.25 per 100,000) and the lowest for females in Spain (0.2/100,000) and males in Estonia (0.2/100.000) (Table 3).

## Discussion

This study focused on drug-overdose suicides (ICD-10, X60-64) in recent years, comparing their prevalence and effects of sex and age in the 16 member countries of the European Commission's 'European Alliance Against Depression' (EAAD) project.

### Sex and country differences

Sex is clearly a major demographic factor in choice of suicide methods [1,10,28]. The recent EAAD study showed that more than a quarter of female suicides involved intentional self-poisoning with drugs, while for males drug self-poisoning accounted for less than 10% [9]. Several factors explain why females ingest drugs for suicidal purposes relatively often. Townsend [15] suggested that females' weaker intention to die may contribute to their high rates of drug overdose. Lester [6] associated the female preference for drug use in suicidal acts with greater concern for bodily appearance after death, and painlessness as one reason for this preference. Shapira [12] stressed males' greater propensity to violence. Regarding availability of means, Moller-Leimkuhler [28] cited the association between females' greater propensity to major depression and availability of prescription drugs, while linking males' higher suicide rate with their more frequent gun ownership.

Despite the sexes' different rates of drug overdose, the present study highlights the similarity of drug self-poisoning rates in most countries and in the aggregate data (1.6 and 1.5 respectively). This similarity is independent of the countries' overall suicide rates and male-to-female ratios. Males' far higher overall suicide rates are due to injury deaths, which were nearly four times higher than in females, while for drug overdoses the male-to-female ratio was very close to 1.

For parasuicide, unlike completed suicide, drug self-poisoning is a high-ranking method for both sexes, indicating its low lethality. The WHO/EURO Multicentre Study on Parasuicide reported that in 14 European countries, 73% of male and 84% of female suicide attempts were due to drug overdoses [5]. The conclusion was that, in the context of overall suicidality, drugs warrant great attention as means. The relatively rarity of fatal outcomes may be due to effective toxicological aid; thus, many suicide attempters using drugs survive and are excluded from suicide statistics. Nevertheless, the WHO/EURO study showed striking differences in drug-overdose rates between countries. This is consistent with the present study's findings on completed suicides.

The country comparisons showed striking variations in sex rates, with implications for preventive policies. In Finland, rates of intentional drug-overdose death are particularly high for both sexes; drug overdoses cause half of the female suicides. Iceland, Scotland, Hungary and Switzerland are other high-risk countries in terms of both sexes' overdose rates, perhaps reflecting the availability of certain drugs, such as antidepressants and psychotropics [29]. Improving depression treatment may lower suicide rates, but prescription drugs can be used for suicidal purposes. More attention should therefore be paid to preventive measures in medical management.

### Subcategories of drugs involved in self-poisoning

Combined data from the countries studied show that, in more than half the cases of male and female suicide by overdose, drugs from the X64 category predominate. Since this category includes 'other and unspecified drugs', data recording is likely to be poor. It may be suspected that doctors issuing death certificates are unwilling to work on detecting drugs taken by the deceased or, in the event of multiple drug use, whether an X64 drug was included. Overall, this category needs refining in terms of, for example, drug combinations, unidentified drugs and biological substances.

Efforts should be made to improve recording systems and investigation procedures, especially in France, Luxembourg, Portugal and Spain, where category X64 drugs are implicated in more than 70% of overdose suicides. The small proportions of X64 drugs in Finland and Scotland reflect careful diagnostics of drugs used for overdoses.

The second most prevalent drug category, X61, is also heterogeneous. Antiepileptic, sedative-hypnotic, anti-parkinsonism and psychotropic drugs are prescribed by different specialists, making it difficult to develop specific strategies to prevent these suicides by systematically limiting access to these medicines. The greatest potential for prevention may be predicted in Finland and Switzerland.

Previous studies, reporting on sex differences in suicide by drug overdose in such countries as Denmark, England and Australia [11,12,21,30,31], suggested that drugs in categories X61 and X60 (non-opioid analgesics etc) predominate as means of suicide. The results of the present study show also that X60 drugs are the most frequently ingested in fatal overdoses in Scotland and England [30], and a much higher rate of self-poisoning with analgesics is reported in the UK than elsewhere in Europe [3,14].

Scotland and Finland, on the other hand, show high rates of suicide using narcotics (X62). Interpretation is difficult; whether these cases are related to drug addicts' deaths not listed as suicides in other countries is unknown. Drug suicide may be difficult to distinguish from accidental death by overdose.

A comparison of the present study's findings with those of the WHO/Euro Multicentre Parasuicide Study [5] shows country-specific similarities in the drugs chosen. For attempted and completed suicides alike, overdose rates for some drugs, such as analgesics (X60) in England and Scotland, and psychotropic drugs (X61) in Finland, are high.

### Age-specific drug overdose

The overall age distribution of suicide rates is not uniform. They peak around age 50 in males and 60 in females. Countries differ, but within each country male and female patterns are highly similar. In Finland and Scotland, high youth suicide rates cause concern [28,32]. Several drug-related studies, subject to limitations of geographical area and size, have highlighted the frequency of drug overdose in the elderly. Since drug overdose is the predominant suicide method among people aged 60 and over, careful prescription is imperative in this age group [2,33].

This study found exceptionally high rates of suicide by drug self-poisoning among the over-65s of both sexes in Switzerland [34]. This partially reflects assisted suicides among the elderly there, and may indicate a need to review the legal basis for assisted suicide, currently offered by two private organisations (EXIT and DIGNITAS) [35]. However, the present study also indicates that both Switzerland and Hungary need to improve suicide prevention among the elderly, perhaps through efforts to enhance their quality of life.

### Prevention

Compared with highly lethal methods, prevention of drug-related suicide across the lifespan has attracted little attention [2], because using drugs is among the least lethal methods [4,10]. Intentional drug overdose, widely used by suicidal individuals, is relatively non-lethal partly because of its distinctive delay between initiation of the suicidal act and death. This delay offers ample scope for prevention: better potential for detection and intervention, and anticipated advances in medical toxicology. Success in these endeavours could turn potentially fatal suicides into suicide attempts. Preventive measures, being far more cost-effective, should nonetheless be given priority.

The number of intentional lethal self-poisoning acts may be underestimated because a sizeable proportion of those who commit them reach hospital alive, but then die from complications. These deaths are not reported as suicides in mortality statistics. The long-term impact of chronic liver damage from paracetamol poisoning is also worth mentioning [14,16,18]. A further factor contributing to underestimation may be that since death by poisoning lacks a dramatic visible outcome (unlike hanging or firearm use), families can conceal the real cause of death.

Preventing drug use in suicidal acts involves careful and appropriate prescription, particularly to treat mental illnesses. This can be achieved through training of GPs, other physicians and pharmacists [36,37]. Overdose safety should be a key criterion for selecting drugs for psychiatric treatment, for example choosing between tricyclic antidepressants and newer, safer antidepressants such as selective serotonin reuptake inhibitors (SSRIs) [38].

Key strategies to reduce self-harm from intentional drug overdose include a public-health approach: informing patients and their families about the dangers of medicines; controlling their availability; and making it easier to dispose of unused tablets [37]. Governments and agencies should be obliged to regulate access to lethal substances, promote training programmes and define international standards. Future development should be based on other countries' best practices, especially those incorporating effective monitoring and evaluation strategies [39]. One such practice may be the 'e-clinic' system being introduced in Estonia, enabling all physicians to share information about their patients' state of health and coordinate prescriptions. However, the e-clinic approach presents real ethical problems regarding extended access to sensitive data.

Contrary to the 'method substitution hypothesis', which suggests that restricting access to some means of suicide results in substitution of alternative methods, numerous studies have demonstrated that such restrictions can reduce the number of completed suicides [36,37,39,40]. Mortality from drug self-poisoning has decreased because of measures to make barbiturates less readily available in Australia [20], England and Wales [12], reduce the size of analgesic packs sold over the counter in the UK [13], improve safety in therapy with psychotropic drugs in Hungary [29], prescribe less toxic medication and smaller amounts of toxic drugs in Denmark [11], and withdraw co-proxamol in Scotland [18]. Mann et al. [36] found that for common methods, restricting means has reduced overall suicide rates, but that it does not entirely discount the method-substitution theory.

### Study strengths and limitations

To our knowledge this is the first time that comparative international data on intentional drug overdose have been analysed in separate categories. The data are not publicly available and were collected separately by each country's project participants. They permitted comparison of the countries by sex, age and categories of drugs used for intentional overdose, and reveal that drug ingestion also causes male suicides. The data could be used to improve suicide diagnostics and suicide prevention programmes. The study revealed also the need to redefine the ICD-10 X60-64 codes in more detail.

This study's limitations include its ecological design and aggregate data level, which rule out detailed investigation of the various drug categories, such as drug names or doses taken in overdoses. The ICD-10 X60-64 codes are clearly an inadequate classification for detailed use and lethality detection. Designing prevention programmes calls for a refined coding system. Further research must focus on specific drugs in national samples.

The study years chosen, 2000-04/05, imposed another limitation. Since the ICD-9 and ICD-10 classifications are not comparable, Ireland -- where ICD-9 was used throughout the study period -- is excluded from the category breakdown of overdose drugs presented in Table 2. England was included after ICD-10 superseded ICD-9 in 2001, and Portugal after its mortality registration system was restructured in 2002.

## Conclusions

Both sexes use drug self-poisoning, a preventable suicide method, equally. Overdose patterns vary widely among the countries, but within them no substantial divergence in sex and age patterns in drug choice are found. The comparative data are relevant for improving suicide registration, designing suicide prevention strategies at national or regional level and learning best practices of drug management in countries where drug overdose is relatively unusual. These data show that drug self-poisoning merits attention from clinical and public-health viewpoints. This requires a more detailed ICD coding system for overdose suicides.

## Competing interests

The authors declare that they have no competing interests.

## Authors' contributions

AV participated in developing the study conception and design, data analysis and interpretation, drafting the article, revising it critically for important intellectual content and final approval of the version to be published. MS, PV, JW and KK participated in data analysis and interpretation, drafting the article, revising it critically for important intellectual content and final approval of the version to be published. EA, MM, TR, RG, CA, GS, CF-C, CC, MK, AS and SR participated in data analysis and interpretation, revising the article critically for important intellectual content and final approval of the version to be published. UH participated in developing the study conception and design, data analysis and interpretation, revising the article critically for important intellectual content and final approval of the version to be published.

## Pre-publication history

The pre-publication history for this paper can be accessed here:

http://www.biomedcentral.com/1471-2458/11/61/prepub
